# Deciphering the roles of neddylation modification in hepatocellular carcinoma: Molecular mechanisms and targeted therapeutics

**DOI:** 10.1016/j.gendis.2024.101483

**Published:** 2024-12-06

**Authors:** Wenxin Wu, Xuanyi Wang, Ruijie Ma, Shuhong Huang, Hongguang Li, Xinxing Lyu

**Affiliations:** a Hospital for Skin Diseases, Shandong First Medical University, Jinan, Shandong 250117, China; b School of Clinical and Basic Medical Sciences, Shandong Provincial Hospital Affiliated to Shandong First Medical University, Jinan, Shandong 250117, China; c Science and Technology Innovation Center, Shandong Provincial Hospital Affiliated to Shandong First Medical University, Jinan, Shandong 250117, China; d Department of Hepatobiliary Surgery, Shandong Provincial Hospital Affiliated to Shandong First Medical University, Jinan, Shandong 250021, China; e Department of Thoracic Surgery, National Cancer Center, National Clinical Research Center for Cancer, Cancer Hospital, Chinese Academy of Medical Sciences and Peking Union Medical College, Beijing 100021, China

**Keywords:** Cancer therapy, Hepatocellular carcinoma, Neddylation, Post-translation modification, Targeted inhibitors

## Abstract

Hepatocellular carcinoma (HCC) is the most prevalent type of malignant liver tumor with high morbidity and mortality and severely threatens human health and life quality. Thus, it is of great significance to investigate the molecular mechanism underlying the pathogenesis of HCC and seek biomarkers for early diagnosis. Neddylation, one of the most conserved post-translational modification types in eukaryotes, plays vital roles in the progression of HCC. During the process of neddylation, NEDD8 is covalently conjugated to its substrate proteins, thereby modulating multiple necessary biological processes. Currently, increasing evidence shows that the aberrant activation of neddylation is positively correlated with the occurrence and development of tumors and the poor clinical prognosis of HCC patients. Based on the current investigations, neddylation modification has been reported to target both the cullins and non-cullin substrates and subsequently affect HCC progression, including the virus infection, malignant transformation, tumor cell proliferation, migration and invasion ability, and tumor microenvironment. Therefore, inhibitors targeting the neddylation cascade have been developed and entered clinical trials, indicating satisfactory anti-HCC treatment effects. This review aims to summarize the latest progress in the molecular mechanism of pathologically aberrant neddylation in HCC, as well as the advances of neddylation-targeted inhibitors as potential drugs for HCC treatment.

## Introduction

Hepatocellular carcinoma (HCC), the predominant form of primary liver cancers, accounts for 90% of all the cases worldwide.^1^ According to the latest statistics, HCC ranks as the sixth most common malignant tumor and the third-leading cause of cancer-associated mortality.^2^^,^^3^ Its high malignancy and poor prognosis pose huge threats to human health. Generally, several risk factors have been considered as the causes of HCC occurrence, including viral hepatitis, long-term alcohol consumption, certain toxins, and obesity. Meanwhile, HCC develops through several well-known cellular processes, such as epithelial–mesenchymal transition, tumor–stromal interaction, tumor microenvironment, cancer stem cell formation, and aging. Currently, based on the stage and clinical heterogeneity of HCC, several potential treatments have been used in clinical practice. For the very early stage of HCC patients, liver resection, local ablative therapy, and liver transplantation are usually the first options to preserve the main functions of the liver. While for the intermediate or advanced stage HCC, systemic therapy will benefit the patient's survival. The first-line drugs such as sorafenib and lenvatinib as well as second-line drugs such as regorafenib and radiotherapy have often been used to improve the survival of advanced patients.^4^ Multiple combination therapies tend to have greater potential for HCC treatment. For instance, the combination of altilizumab and bevacizumab immunotherapy is more effective than sorafenib in the treatment of advanced inoperable liver cancer. Another two immune-based regimens (pembrolizumab and navulizumab in combination with ibritumomab) have entered the phase II clinical trial stage. Single-agent immune checkpoint inhibitor combined with anti-VEGF antibody therapy targeting PD1, and combination therapy of immune checkpoint inhibitor and tyrosine kinase inhibitor have entered phase II–III clinical trial stage, and clinical trials of novel drugs targeting cancer stem cells (such as icariin and DKN-01) are also in progress. Currently, all the attempts have shown to be beneficial to the prognosis of HCC patients.^5^ Even so, HCC patients still suffer high lethality and low five-year survival rates compared with many other cancers. The lack of comprehensive understanding of the detailed molecular pathogenesis still severely impeded the development of effective therapeutic therapeutics for HCC. Thus, it is urgent to explore the more specific and effective targets for HCC treatment.

Post-translational modification of proteins greatly extends their functions and facilitates cells to adapt to various physiological and pathological conditions by the changes in the state of charge, stability, and conformation of the targeted proteins. To date, no less than 450 unique protein modifications have been identified. Among them, ubiquitin and ubiquitin-like modifications have been reported to engage in multiple important cellular processes, such as protein activation and degradation, cellular growth, proliferation, and differentiation, which are significantly associated with tumorigenesis. Based on their evolutionary sequence traits, the ubiquitin-like proteins (Ubls) are categorized as NEDD8, SUMO, ISG15, FUB1, FAT10, Atg8, Atg12, Urm1, and UFM1. Among them, NEDD8 (neural precursor cell expressed developmentally down-regulated protein 8), which mediates the post-translational modification called neddylation, was first reported to activate the cullin RING E3 ligases and some other non-cullin functional proteins. The dysfunction of neddylation is closely associated with various disorders of the human body.^6^ In decades, a growing body of evidence validates that the alteration of neddylation greatly affects the progress of HCC. In this review, we described the effects of neddylation on HCC through the modification of NEDD8 on cullin and non-cullin family and how to affect the responding cellular processes, and finally discussed the current available neddylation-targeted inhibitors and envisioned their therapeutic potential in HCC treatment.

## The structure of NEDD8 molecule

NEDD8, one of the most popular Ubls shows universal expression in eukaryotic organisms and is highly conserved among species. Besides, NEDD8 shows high sequence similarity with ubiquitin (60% amino acid sequence similarity and 80% homology).^7^ Distinct from the cytoplasmic expression of ubiquitin, NEDD8 predominantly localizes within the nuclei, as reported in cardiac and skeletal muscle cells.^8^ The distinct subcellular localization indicated their different cellular functions. In addition, the precursor of NEDD8, comprising 81 amino acid residues, needs to be hydrolyzed by its last five amino acids to mature. The mature NEDD8 possesses a conserved secondary structure resembling that of ubiquitin. Its architecture encompasses a globular domain (residues 1–72) adopting the folding pattern observed in ubiquitin and a C-terminal tail composed of residues 73–76, where Gly76 is required to be hydrolyzed to enter the neddylation process.^9^ The globular head comprises the primary secondary structural elements: a 3.5-turn α helix (α1, residues 23–34), a short 310-helix (α2, residues 56–59), and a five-chain mixed β sheet. The inner chain composed of residues 1–7 (β1) and 64–72 (β5) is parallel to each other, while the outer chain composed of residues 10–17 (β2), 40–45 (β3), and 48–50 (β4) is antiparallel to the inner chain, and the loop formed between the β1 and β2 chains is important for the modification of cullin-RING E3 ubiquitin ligases (CRLs) by NEDD8. The carbonyl carbon and amido-nitro group between the β-lamellar chains are stabilized by hydrogen bonds. The Patches of Ile44, Leu8, and Val70 exposed on NEDD8's β chain surface interact with UBA3 to activate NEDD8's C-terminal during deneddylation. Similar to ubiquitin, the C-terminal tail region of NEDD8 is an independent part of its globular domain that does not interact with other regions but contains seven necessary residues. Ending in a Gly–Gly sequence, the C-terminal region plays a vital role in modification processes and adopts a distinct extended structure when interacting with neddylation and deneddylation enzymes.^10^

In addition, the functions of NEDD8 are also regulated by some other post-translational modification molecules. For instance, NEDD8 molecule can be modified by phosphorylation. As reported, the S65 site of NEDD8 can be phosphorylated by PINK1, as the same phosphorylated site of Ub. S65 phosphorylation dynamically changes the secondary structure of NEDD8 between the “relaxed state” (unmodified mode) and “retracted state” (phosphorylated mode) with the C-Terminal β5-strand slippage, and subsequent remodeling of corresponding hydrogen bridges.^11^ The phosphorylated NEDD8 exhibits higher affinity to HSP70 and increases HSP70A activity.^12^ In addition, NEDD8 could also be modified by Ub or Ubl molecules and then form diverse polymers, such as poly-NEDD8, hybrid NEDD8-ubiquitin, and NEDD8-SUMO-2 chain. Similar to the ubiquitin molecule, such modifications mainly occur at the K6/K11/K22/K27/K33/K48/K54 lysine residues of NEDD8 and are executed by the canonical and atypical neddylation processes. The polyNEDD8 and hybrid NEDD8 chains enhanced the crosstalk between the neddylation and other Ub/Ubls pathways. The dynamic balance between mono-NEDD8 and poly-NEDD8 is essential for multiple cellular processes, including protein stability maintenance, apoptosis, and DNA damage repair under stress conditions.^13^ Meanwhile, the lysine residues in NEDD8 were found to be subject to acetylation. P300 functions as a position-specific acetyltransferase for NEDD8 acetylation at K11, and such acetylated NEDD8 limits its interaction with E2 enzymes UBE2M and UBE2F to suppress the neddylation pathway.^14^ Therefore, post-translational modifications of NEDD8 itself play a vital role in the whole neddylation pathway regulation.

## The neddylation cascade

### NEDD8 maturation

The initial translation product of NEDD8 is a precursor, which needs to hydrolyze the last five amino acids (GGLRQ) at its C-terminal and then becomes the mature NEDD8. The exposed di-Gly motif at the C-terminal is necessary for the neddylation initiation. Generally, C-terminal resection of NEDD8 is usually mediated by two specific cysteine proteases, ubiquitin C-terminal hydrolase3 (UCHL3) and deneddylase 1 (DEN1). UCHL3 processes both the NEDD8 and Ubiquitin. NEDP1 (DEN1 or SENP8) has dual roles of specifically processing the NEDD8 precursor C-terminal resection, as well as deconjugating the neddylation from its substrate proteins. In mammalian cells, the deconjugated NEDD8 can also re-enter the neddylation cycle.^15^

### Canonical neddylation cascade system

Similar to ubiquitylation and other Ubl post-translational modifications, the neddylation process goes through a three-step sequential enzymatic cascade, which was mediated by E1, E2, and E3 enzymes. Despite the structural similarity between ubiquitin and NEDD8, the cascade of neddylation has its own specific E1 and E2 enzymes.

The mature NEDD8 molecule needs to be activated by NEDD8-activating enzyme E1 (NAE), a heterodimer composed of NAE1 (APPBP1) and UBA3.^16^ The overall structure indicates three functional domains in NAE, including the adenylation site, cysteine transthiosylation domain, and ubiquitin folding domain. Firstly, the adenylation site of UBA3 binds to ATP and NEDD8 to catalyze the adenylation of the carboxylate terminal Gly76 residue of NEDD8 to form NEDD8-AMP intermediate in the presence of Mg^2+^. Subsequently, Cys216 residue of UBA3 reacts with NEDD8-AMP intermediate and then replaces the AMP to form NEDD8-NAE by forming a thioester bond.^17^ After that, the NEDD8-NAE heterodimer binds to a second NEDD8 to the adenylation domain to form a NEDD8-NAE-NEDD8-AMP complex, resulting in a conformational rearrangement of ubiquitin folding domain. From the investigation in a large cohort of HCC patients, the NAE1 mRNA was found to significantly increase at the transcriptional and protein levels, and NAE1 expression was significantly correlated with poor prognosis of HCC.^18^ The RNA-binding protein RBM15 could bind to NAE1 mRNA and maintain its stability in cardiomyocytes, while RBM15 was also highly expressed in HCC and positively associated with the poor outcomes of patients.^19^^,^^20^ Thus, we proposed that RBM15 probably also stabilizes NAE1 mRNA in HCC development. Besides, lncRNA CYP1B1-AS1 also directly interacts with NAE1 to reduce the targeted protein neddylation in cancer cells.^21^ Therefore, the increased expression of NAE1 plays a vital role in the overaction of neddylation in HCC.

Two NEDD8-conjugating enzymes (E2s), UBE2M and UBE2F, have been identified in the neddylation cascade. Both the two E2s are capable of transferring NEDD8 from the active site cysteine residue of E1 to the active site cysteine of E2 catalyzed by a transthiolation reaction. The crystal structure of the intermediate complex indicates that NEDD8-activating enzyme E1 forms a large central groove to cradle NEDD8 (2)-MgATP-E2 together, in which the groove formed by the α3 helix of UBE2M is wrapped around the β1/β2 loop of NEDD8 and links to NEDD8 and E2 directly.^22^ Through transesterification, the activated NEDD8 molecule dissociates from the activating enzyme E1 UBA3 subunit and forms a new thiol ester bond with the NEDD8 binding enzyme E2. UBE2M and UBE2F specifically bind to the double NEDD8 loaded NAE, which leads to the E1 cycling back and forth between the double and single NEDD8-loaded forms. In HCC, both two E2 mRNAs were up-regulated, and the HCC patients with higher UBE2M expression indicate poorer overall survival and recurrence-free survival.^18^^,^^23^ In cancer cells, the up-regulated UBE2M mRNA was found to be stress-inducible by hypoxia and mitogen stimulation via HIF-1α and AP1. With these stress stimuli, UBE2M works as a ubiquitin E2 for Parkin/DJ-1 E3 and subsequently degrades UBE2F. While under normal conditions, UBE2M acts as a neddylation E2 to activate Cul3/keap1 E3 and to degrade UBE2F. Both dual roles of UBE2M negatively regulate the stability of UBE2F.^24^ Besides, the enzyme activities of both E2s are also regulated by post-translational modification, and their N-terminal Met acetylation was reported to contribute to the complex formation of CUL-DCNL-E2 and subsequently to selectively activate NEDD8 ligation to cullins.^25^ Meanwhile, the E2 activity can be regulated by interacting with glycyl-tRNA synthetase, which acts as a chaperone to protect the stabilization of activated NEDD8-UBE2M before it reaches the neddylated targets.^26^ Therefore, all these strategies promote the E2 expression and/or stabilization to promote the neddylation process, which can also be considered as potential therapeutic targets for cancers.

Next, the NEDD8 is transferred from E2 onto the specific protein substrates via the distinct NEDD8 E3 ligases. NEDD8 E3 ligase catalyzes an isopeptide linkage between the carboxyl-terminal glycine Gly76 of NEDD8 and the lysine residue of the target molecules. At present, various kinds of NEDD8 E3 ligases have been identified. Except for the DCN1-LIKE proteins, other NEDD8 E3 ligases currently reported belong to the RING subclass, such as Ring Box Protein RBX1 and RBX2, mouse double minute 2 (MDM2), Casitas B-lineage lymphoma (c-CBL), and F-box protein 11 (FBXO11). Among them, RBX1/RBX2 are the two best-studied NEDD8 E3 ligases and mainly catalyze the neddylation of cullin family proteins. Most NEDD8 E3 ligases also show ubiquitin E3 ligase activity. Thus, the dual functions of these E3 ligase enzymes greatly extend the post-translational modification types of target proteins.

Neddylation is a reversible process and the dissociated NEDD8 can also come back into the neddylation cascade. The dissociation process is catalyzed by the bifunctional enzyme NEDP1 and the specific CSN5, which serves as the fifth subunit of zinc metalloproteinase COP9 (CSN) with a total of 8 subunits and exhibits catalytic activity. CSN5 exhibits higher catalytic activity for deneddylation compared with NEDP1. Specifically, CSN5 mainly facilitates deneddylation in the cullin family, while NEDP1 mediates deneddylation in other protein substrates. Then, the dissociated NEDD8 molecule can be recycled again into a new neddylation process and continues to conjugate to the substrates.^27^

### Atypical neddylation processes

In addition to the canonical neddylation process as discussed above, the NEDD8 conjugating to substrates can also occur in a neddylation conjugating cascade-independent manner, termed atypical neddylation. Atypical neddylation mainly relies on a ubiquitination-conjugating system, in which NEDD8 is nonspecifically activated by ubiquitin E1 enzyme UBA1 when NEDD8 is overexpressed in cells, and then activated NEDD8 will be transferred to ubiquitin-specific E2 enzymes and finally conjugated to the ubiquitylation substrates. Interestingly, profiles of proteome-wide NEDD8 modified substrates exhibit distinct proteins in various cellular pathways by virtue of the canonical and atypical neddylation processes. Atypical neddylation predominantly occurs under stress conditions, especially proteotoxic stress conditions, and functions to form poly-NEDD8 or hybrid NEDD8-ubiquitin and NEDD8-SUMO complexes.^28^ The substrates of atypical neddylation are mainly the ribosomal proteins, causing the aggregation of ribosomal proteins in the nucleus, resulting in a defense state where the ubiquitination-proteasome system has a reduced load of misfolded proteins during stress.^29^ In such a process, HUWE1, identified as a key ubiquitin E3 ligase, promotes the atypical neddylation modification to inhibit target protein degradation and promotes misfolded protein aggregation under proteotoxic stress conditions. The interferon-inducible protein NEDD8 ultimate buster 1 long (NUB1L) inhibits atypical neddylation by promoting NEDD8 degradation, which promotes proteasomal degradation of misfolded proteins.^30^ Therefore, atypical neddylation is an important regulator of protein homeostasis under stress conditions.

### Neddylation drives liver disease to induce the development of HCC

**Figure 1:**
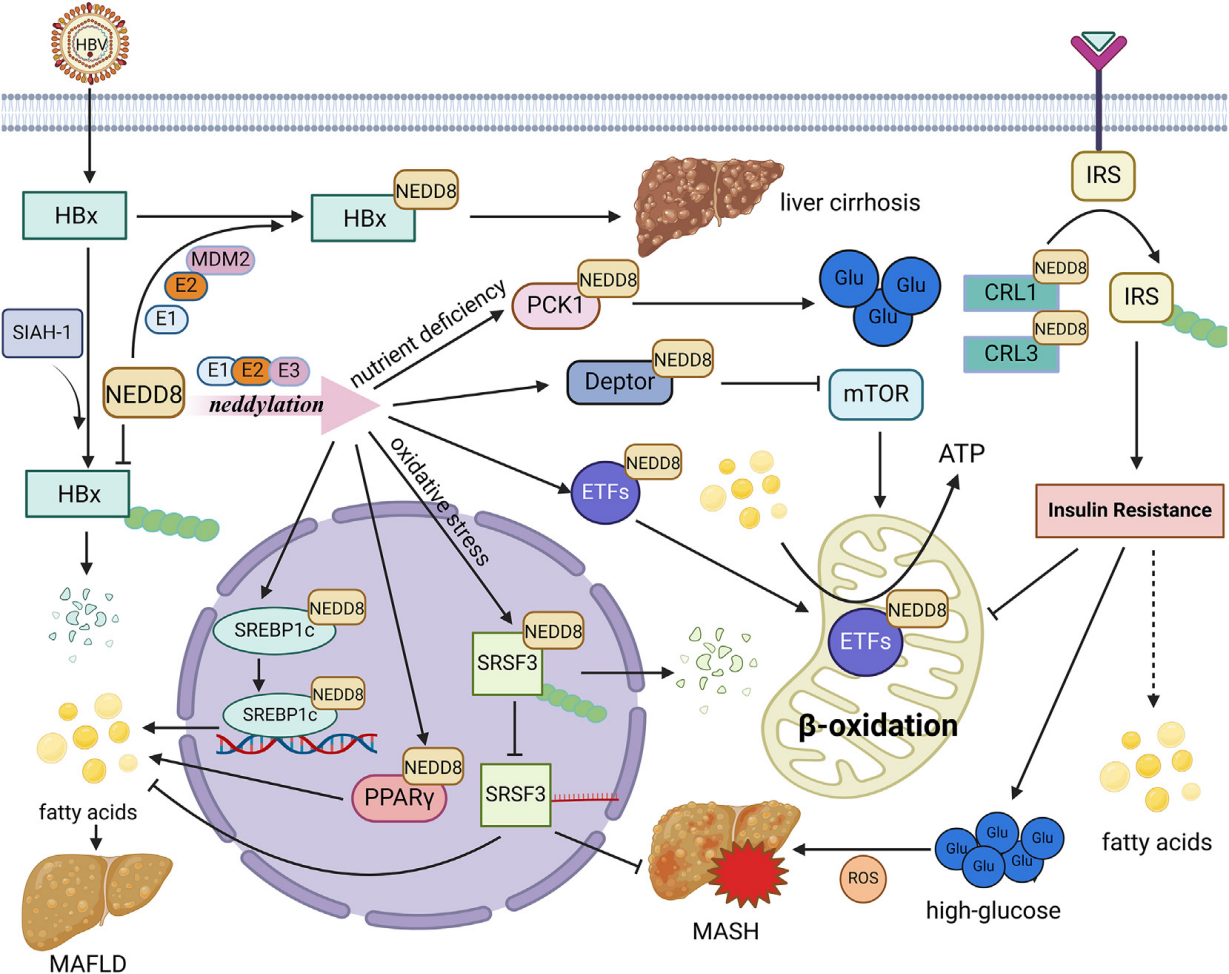
Neddylation drives liver disease to induce the development of hepatocellular carcinoma. (i) HBx produced from HBV infection is modified by NEDD8 to avoid degradation by SIAH-1, retaining its activity and promoting hepatitis development. (ii) Neddylated forms of SREBP-1c and PPARγ increase their activities, affecting the transcription of genes related to lipid metabolism, promoting the synthesis of fatty acids, and leading to intrahepatic steatosis and the development of metabolic-associated fatty liver disease. SRSF3 is degraded after neddylation, resulting in its diminished regulation of metabolic homeostasis, and oxidative stress induced by hepatic steatosis further promotes SRSF degradation. (iii) Neddylated Deptor and ETFs have enhanced their activities and affected fatty acid metabolism. (iv) CRL1 and CRL3 target insulin receptor substrate (IRS) for ubiquitination degradation, leading to insulin signaling in hepatocytes, which affects cellular lipid metabolism and glucose metabolism. All the risks associated with liver diseases are also closely related to hepatocellular carcinoma development. HBx, hepatitis B virus X protein; HBV, hepatitis B virus; NEDD8, neural precursor cell expressed developmentally down-regulated protein 8; SIAH-1, Siah E3 ubiquitin protein ligase 1; SREBP1c, sterol regulatory element binding protein 1c; PPARγ, peroxisome proliferator-activated receptor γ; SRSF3, serine-rich splicing factor 3; ETFs, electron transfer flavoproteins; Deptor, EP-domain containing mTOR-interacting protein; CRL, cullin-RING E3 ubiquitin ligase.

Epidemiological investigation has revealed that patients with liver diseases, such as virus infection-related liver disease and alcohol-associated and metabolic dysfunction-associated steatotic liver disease, are susceptible to HCC occurrence.^31^^,^^32^ Emerging evidence indicates the aberrant activation of the neddylation pathway in these liver diseases, which suggests the possible functions of neddylation in the transformation of these liver diseases to HCC.^33^ The interventions in the neddylation pathway might potentially halt the progression from earlier stages of liver disease to full-blown HCC (Fig. 1).

According to the data from the global burden of disease study, 69.5% of HCC cases were related to virus infection (hepatitis B virus/HBV and hepatitis C virus/HCV).^34^ Thus, HBV and HCV infection-driving chronic hepatitis is associated with the development of HCC. In the HBV-infected hepatocyte cells, HBV-encoded hepatitis B virus *X* protein (HBx) is neddylated by HDM2, which then stabilizes HBx by preventing Siah E3 ubiquitin protein ligase 1 (SIAH-1)-mediated ubiquitin–proteasome degradation.^35^ Both NEDD8 knockdown and MLN4924 treatment inhibit HBV replication in hepatocyte cells.^35^^,^^36^ Therefore, the overactivated neddylation might increase the risk of HCC occurrence by promoting HBV infection.

The metabolic-associated fatty liver disease is characterized by excessive triglyceride accumulation in hepatocytes accompanied by advanced fibrosis, which eventually leads to HCC.^37^ Hepatic global neddylation was induced in metabolic-associated fatty liver disease patients and animal models. Neddylation modification in hepatocytes was found to participate in lipid metabolism and be involved in the progression of metabolic-associated fatty liver disease. Some proteins are involved in hepatic lipid metabolisms, such as electron transfer flavoproteins (ETFs), serine-rich splicing factor 3 (SRSF3), peroxisome proliferator-activated receptor γ (PPARγ), EP-domain containing mTOR-interacting protein (Deptor), and sterol regulatory element binding protein 1c (SREBP1c); they are directly modified by NEDD8 and affect lipid degradation and the following hepatic steatosis.^38^, ^39^, ^40^, ^41^, ^42^, ^43^ The hepatic neddylation process is a potential therapeutic target to alleviate metabolic-associated fatty liver disease and prevent HCC occurrence.

Besides the lipid metabolism, neddylation in the liver regulates glucose homeostasis, which is closely related to tumorigenesis.^43^ The level of neddylation in the liver was modulated by nutrients. The elevated neddylation in type 2 diabetes mellitus patients is a high risk for the development of liver cirrhosis.^44^ Reduced expression of neddylation E3 ligases cullin-1 and cullin-3 or MLN4924 treatment stabilized hepatic insulin receptor substrate to decrease blood glucose.^45^ Besides, inhibition of hepatic neddylation can decrease gluconeogenic capacity and hyperglycemic actions by targeting phosphoenolpyruvate carboxykinase 1 (PCK1).^43^ Therefore, the finely tuned hepatic glucose metabolism by neddylation would prevent the occurrence of steatotic liver disease and HCC.^46^^,^^47^

Given that liver diseases are susceptible to liver cancers, especially for HCC, preventive treatment of liver diseases is a much more effective clinical strategy than liver cancer treatment. Thus, the interventions of the neddylation pathway potentially halt the progression from earlier stages of liver disease to full-blown HCC.

### The roles of neddylation in HCC progression

**Figure 2:**
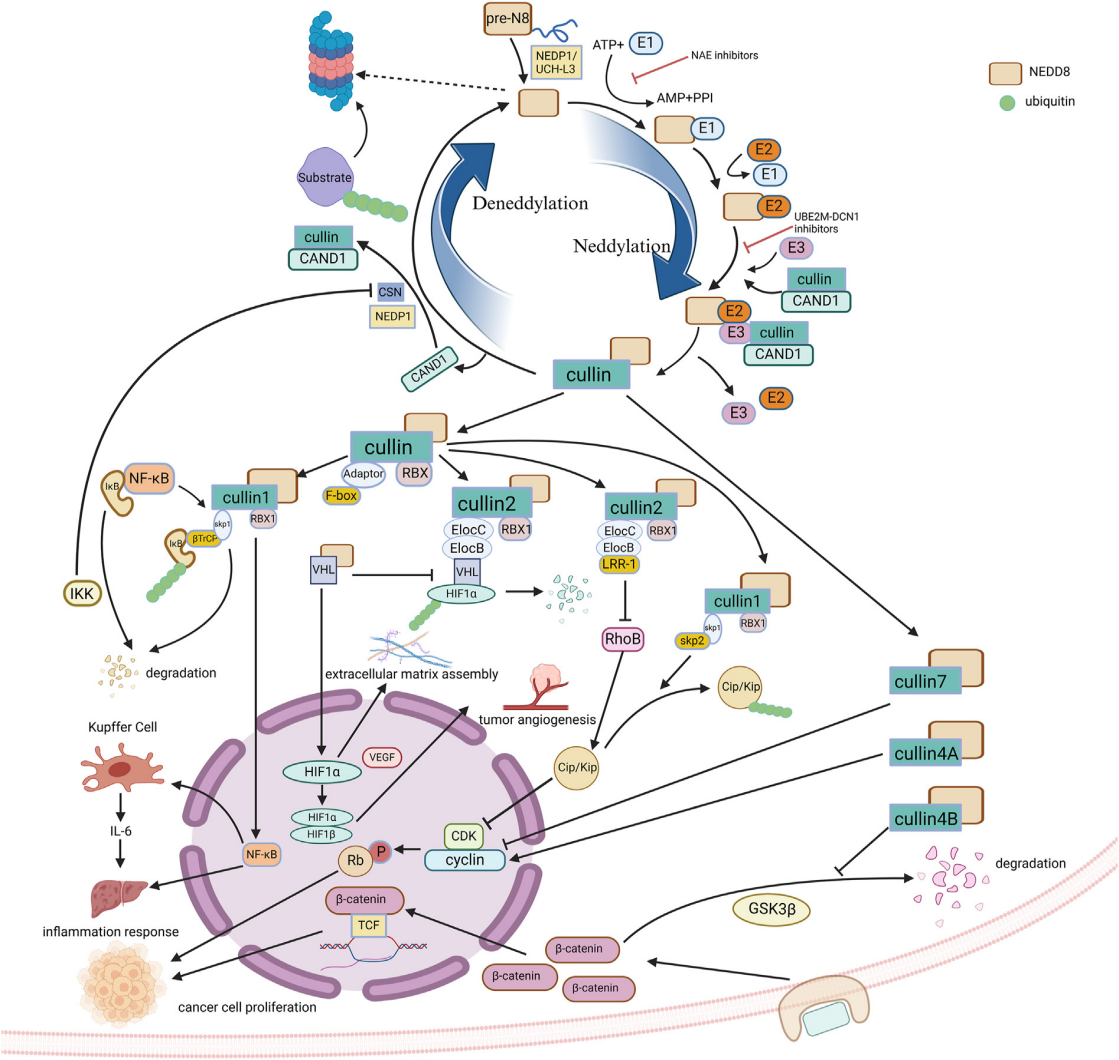
Schematic representation of the neddylation process of cullin proteins and their regulatory roles in cell signaling in hepatocellular carcinoma. Mature NEDD8 activates the assembly of CRLs via modifying cullins with neddylation-cascade enzymes, and therefore alters the cullin conformation and detaches CAND1 from the cullin molecule. Detailed cellular processes are shown as follows: (i) SCF^βTrCP^ degrades IκB proteins by ubiquitination to activate NF-κB, and then promotes the downstream of inflammatory responses. (ii) SCF-Skp2, composed of Skp2, Skp1, CUL1 and RBX1, degrades Cip/Kip by ubiquitination, resulting in the loss of negative regulation of CDKs, which leads to the overactivation of cyclin and the dysregulation of the cell cycle. (iii) CRL2-LRR-1 mediates RhoB inactivation, leading to a decrease in downstream Cip/Kip and promoting cyclin activation. (iv) VHL–HIF–1α pathway. VHL is involved in the formation of the ubiquitin ligase ECV, which targets HIF-1α for ubiquitination, but after VHL is modified by NEDD8, it is no longer involved in the formation of ECV due to a conformational change, thus stabilizing HIF-α, which forms a transcription factor with HIF-β to mediate the downstream VEGF to promote tumor angiogenesis. (v) Neddylated CUL4B activates downstream signaling of the Wnt/β-catenin pathway by protecting downstream β-catenin signaling from GSK3β-mediated degradation. (vi) Neddylated CUL4A and neddylated CUL7 affect cell proliferation by regulating cyclin. NEDD8, neural precursor cell expressed developmentally down-regulated protein 8; CRL, cullin-RING E3 ubiquitin ligase; CAND1, cullin associated and neddylation dissociated 1; SCF, SKP1-CUL1-F-box; NF-κB, nuclear factor-κB; CDKs, cyclin-dependent kinases; VHL, Von Hippel-Lindau; HIF, hypoxia-inducible factor; ECV, elongin B/C–CUL2–VHL; VEGF, vascular endothelial growth factor; GSK3β, glycogen synthase kinase 3β.

The neddylation pathway has been reported to be aberrantly activated in HCC patients, and the up-regulation of NEDD8 leads to a high risk for overall survival and recurrence-free survival of HCC patients.^18^ Functionally, neddylation modification usually emerges as activating signaling by changing the properties of its target substrate, including protein structures, stability and location, and interaction, and engages in promoting the HCC oncogenesis and development via affecting cell proliferation, cell cycle, DNA replication, and apoptosis.^48^^,^^49^ Generally, the substrates of neddylation are often categorized into two groups, the cullin family of proteins and non-cullin proteins. Cullin proteins are essential for the activation of CRLs, and non-cullin protein substrates include multiple signaling molecules, such as HBx, SREBP-1c, HuR, LKB1, AKT, pVHL, TGFβⅡ, and EGFR. Herein, we elaborately summarized the manners of neddylation modification affecting cell signaling to promote the development of HCC (Fig. 2 and Table 1).

**Table 1:** Neddylation substrates and their functions in hepatocellular carcinoma.

Type	Substrates	E3 ligases	Function of neddylation
Cullin family	Cullin1	RBX1	Inflammatory response and fibrosis of hepatocytes and proliferation of HCC cells, accumulation of DEPTOR and promoting drug resistance in MLN4924
	Cullin2	RBX1	HCC cell migration and invasion, accumulation of HIF-1α and promoting drug resistance in MLN4924
	Cullin4A	RBX1	EMT in HCC cells and HCC migration and invasion
	Cullin4B	RBX1	HCC cell proliferation
	Cullin7	RBX1	EMT in HCC cells and HCC migration and invasion
Non-Cullin family	LKB1	__	Adaption of hepatocellular carcinoma cells to hypoxic stress environment
	AKT	__	Adaption of hepatocellular carcinoma cells to hypoxic stress environment
	TGFβⅡ	c-CBL	Liver fibrosis
	EGFR	c-CBL	Resistance to tyrosine kinase inhibitors
	SREBP-1c	__	Non-alcoholic fatty liver disease (NAFLD)
	VHL	MDM2	The process of HCC extracellular matrix assembly
	HBx	MDM2	Enhancement of HBV gene transcriptional activity
	HuR	MDM2	Inflammatory response and fibrosis in hepatocytes and HCC transformation process

RBX1: Ring-Box 1, an essential component of SCF (Skp1/Cullins/F-box) E3 ubiquitin ligases; HCC: Hepatic Cell Carcinoma; DEPTOR: DEP domain-containing mTOR interacting protein, a natural mTOR inhibitor; EMT: Epithelial–Mesenchymal Transition; LKB1: Liver Kinase B1, a protein kinase; AKT: a serine/threonine kinase; TGFβⅡ: Transforming Trowth Factor-β receptor type Ⅱ; c-CBL: Casitas B lymphoma, an E3 ubiquitin ligase; EGFR: Epidermal growth factor receptor; SREBP-1c:Sterol regulatory element-binding protein 1, a key transcription factor; VHL: Von Hippel-Lindau Tumor Suppressor; MDM2: murine double minute 2, a E3 ligase; HBx: Hepatitis B virus *X* protein; HuR: Human antigen R, a RNA binding protein.

### The roles of cullin protein neddylation in HCC development

Cullin family proteins function as molecular scaffolds of CRLs and are the first identified and most famous substrates of neddylation.^50^ Structurally, the N-terminus of cullin binds adapter proteins and substrate recognition subunits, and its C-terminus binds to the RING proteins Rbx1 or Rbx2, assembling as CRLs.^51^ CRLs mediate about 20 % ubiquitin protein degradation via the ubiquitin-proteasome system. Currently identified members of the cullin family include cullin-1, -2, -3, -4A, -4B, −5, −7, and −9.^52^ NEDD8 enhances E3 ligase activity in CRLs. It causes a conformational change in cullin protein, enhances the binding of Rbx1 and Rbx2 to ubiquitin E2 ligase, and shortens the distance between E2 and the substrate recognition subunit, which in turn brings ubiquitin in close proximity to the target protein and promotes the extension of the ubiquitin chain; besides, the NEDD8 molecule enables the negative CRL regulator CAND1 (cullin associated and neddylation dissociated 1) to be dissociated from the cullin molecule to facilitate the binding of cullin to junction proteins and substrate recognition subunits, which in turn facilitates the assembly of functional CRLs, acting as E3 ubiquitinates to promote ubiquitylation of the substrate and to regulate the cellular activities. NEDD8 enhances intrinsic CRL ubiquitin activity and prevents the binding of CRL to the inhibitor CAND1, allowing CRL to function. CRL activation has been reported to be involved in tumor genesis and development, especially in liver cancers.^53^

### Cullin-1

Cullin-1 is the scaffold of the SCF (SKP1-CUL1-F-box) E3 ubiquitin ligase complex. SCF is the largest family of E3 ligases, and more than 110 F-boxes mediate the ubiquitination of their corresponding substrates and regulate multiple cellular progressions. SCF mediates more than 20 % ubiquitination of proteins in the ubiquitin-proteasome system and is therefore involved in the procession of cell cycle, tumorigenesis, and cellular signaling transduction.^50^ Neddylation modification of cullin-1 is essential for the activation of the SCF complex. In HCC, SCF level is elevated due to the increased level of neddylated cullin-1, which engages in the progression of liver fibrosis and the regulation of the HCC cell cycle.

Nuclear factor-κB (NF-κB) plays a vital role in the transition from hepatic injury and fibrosis to HCC. β-TrCP is a member of the F-box structural domain family and forms SCF^β−TrCP^ in complex with NEDD8-Cul1, and regulates the NF-κB signaling pathway in HCC. SCF^βTrCP^ acts as an IKK (IκB kinase) to achieve ubiquitylated degradation of IκB proteins, activating the NF-κB pathway, which promotes hepatic fibrosis by sustaining the LTα/β inflammatory response. Meanwhile, activated Kupffer cells also secrete a panel of inflammatory cytokines including IL-6 to promote HCC in an IKK/NF-κB-dependent manner.^54^ IL-6 has an inhibitory effect on immune cells and promotes immune evasion of HCC cells. Neddylation of CUL1 affects the activity of SCF^βTrCP^, which then elevates the activation level of the NF-κB signaling pathway and induces the expression of downstream genes.^55^^,^^56^

In addition to SCF^β−TrCP^, SCF^skp2^, comprising another substrate-recognition component Skp2, plays an important role in the development of HCC. SCF^skp2^ mediated the stability of Cip/Kip proteins, consisting of cyclin-dependent kinase inhibitors. The mammalian CiP/KiP family includes three proteins, p21 (Cip1/WAF1), p27 (Kip1), and p57 (Kip2). In HCC cells, cullin-1 was overexpressed and led to an increase in the level of ubiquitin ligase SCF^skp2^. Then, SCF^skp2^ bound to Cip/Kip proteins, causing them to be degraded by ubiquitination, which led to a decrease in the negative regulation of the cyclin-dependent kinases. Without the inhibitory effect on the cell cycle, the overactivated cyclin-dependent kinases would cause the uncontrolled proliferation of HCC cells.^57^^,^^58^

### Cullin-2

Cullin-2 forms an E3 ligase with Rbx1, Elongin C, Elongin B, and a variable substrate-recognition subunit (SRS), in which SRS determines the substrate specificity of the CRL complex and thus its associated cellular functions.^59^ In HCC, neddylation mainly functions through affecting LRR-1-type and Von Hippel-Lindau (VHL)-type CUL2-Rbx1 E3 based on the different types of SRS proteins. NEDD8 modifies cullin-2 and promotes LRR-1-type CUL2-Rbx1 E3 targeting RhoB for ubiquitination, which reduces the expression of the downstream tumor suppressor genes p21 and p27 to promote hepatocarcinogenesis.^60^ Rather than the canonical role of p21 in the nucleus inhibiting cyclin-dependent kinase, CRL2^LRR−1^ mediates the degradation of cytoplasmic p21 and then limits cell motility in cancer cells.^61^ VHL-type CUL2-Rbx1 E3 targets the HIF-1α subunit for ubiquitination-mediated degradation. NEDD8 can simultaneously modify both cullin-2 and VHL proteins. Clinical results indicate that negative expression of VHL shows a worse prognosis for HCC patients.^62^ VHL exhibits dual roles of HIF-1α destruction and fibronectin extracellular matrix assembly, in which the two functions are exclusive and the selectivity is predominantly mediated by neddylation. The non-neddylated VHL would promote its association with CUL2 and then contribute to the ubiquitination modification of HIF-α to promote proteasomal degradation.^63^ The abnormal accumulation of HIF-α forms a heterodimeric transcription factor with HIF-β to promote tumor angiogenesis via activating the expression of vascular endothelial growth factor. Neddylated VHL selectively binds to fibronectin and promotes the fibronectin extracellular matrix assembly process.^64^^,^^65^

### Cullin-4

The CUL 4A protein binds to ring box protein 1 (Rbx1) and DNA damage-binding protein 1 (DDB1) to form DDB1-CUL4-RBX1 E3 ubiquitin ligase. CUL4A expression is up-regulated in HCC.^66^ Increased activity of CUL4A affects cell cycle regulation through dysregulation of cyclin A, cyclin B1, and cyclin *E*, leading to malignant growth of HCC. CUL4A also promotes epithelial–mesenchymal transition and migration of HCC, but the exact molecular mechanism of CUL4A's role in HCC is unknown to date^.^ Some statistical analyses showed that CUL4A expression was negatively correlated with tumor differentiation grade and patient survival, and positively correlated with the extent of lymphatic and venous infiltration.^67^

Cullin-4B acts as the scaffold part of the cullin-4B-Ring E3 ligase and has been less studied compared with cullin-4A. NEDD8 modifies cullin-4B at elevated levels in HCC.^68^ It has been found that CUL4B transgenic mice have high liver cell proliferation compared with normal mice and exhibit compensatory hepatocyte proliferation after dimethylnitrosamine-inducing liver injury.^69^ CUL4B activates the Wnt/β-catenin signaling pathway by protecting β-catenin from glycogen synthase kinase 3β-mediated degradation, which promotes the development of HCC.^70^

### Cullin-7

Cullin-7 is closely associated with HCC.^71^ Cullin-7 forms CUL7-Ring ligase 7 (CRL7) together with Rbx1, the junction protein Skp1, and the F-box protein Fbxw8 (also known as Fbx29). In the development of HCC, elevated levels of neddylated cullin-7 and aberrant activation of cullin-7 promote epithelial–mesenchymal transition in HCC cells^,^ leading to HCC cell migration and invasion.^72^^,^^73^ In addition, the high expression of cullin-7 leads to a decrease in cyclin D1 expression and influences cell proliferation, which is potentially involved in the metabolic syndrome-induced HCC.

Although the roles of neddylation modification of other cullin family members, including cullin-3, -5, and -9, have not been adequately reported to engage in the progression of HCC, their expression levels are closely related to the occurrence of HCC and need to be further investigated.^74^, ^75^, ^76^

### Roles of non-cullin family protein neddylation in HCC

**Figure 3:**
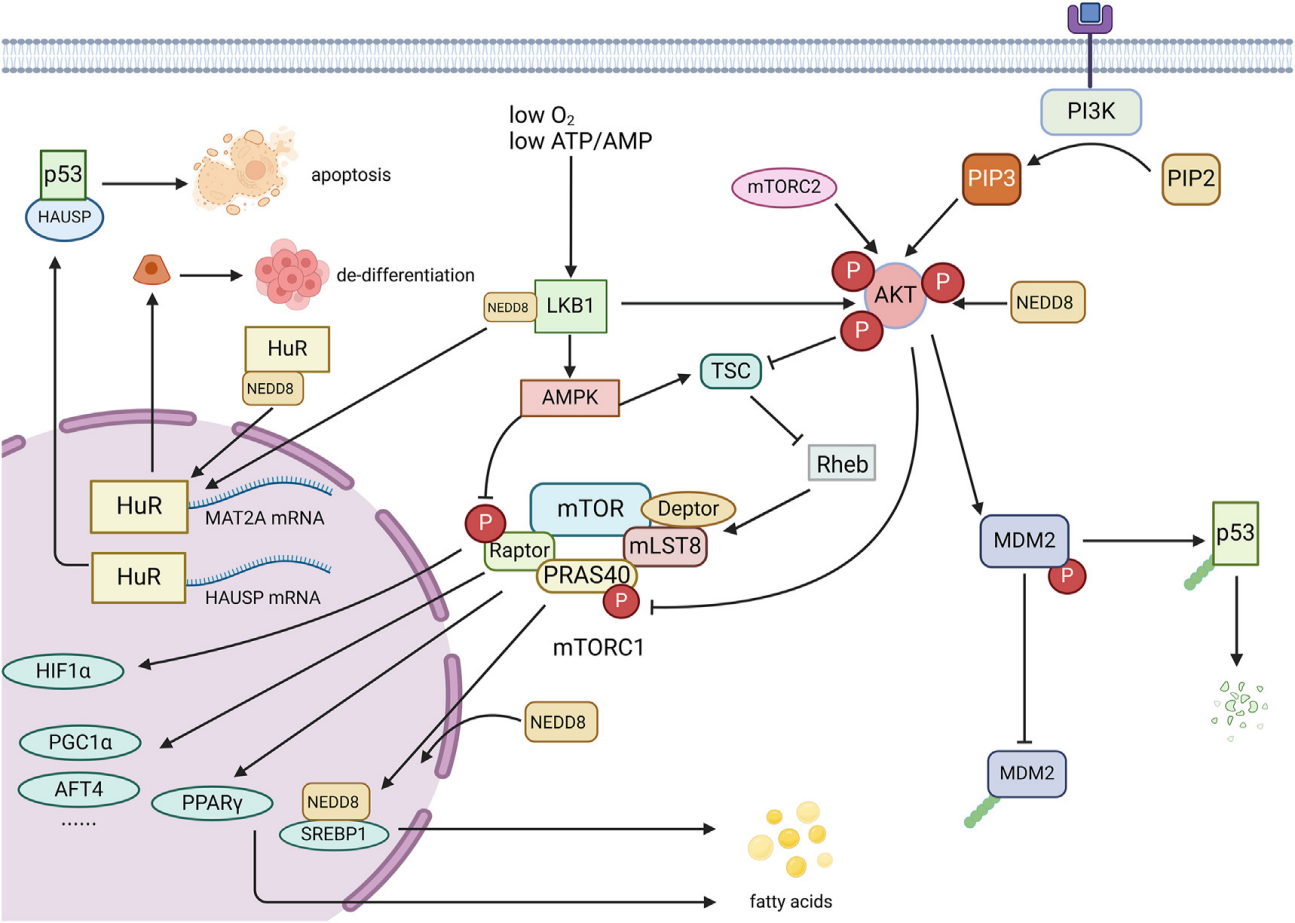
Neddylation of non-cullin substrate-associated signaling pathways in hepatocellular carcinoma. (i) LKB1-AMPK/AKT pathway. Under hypoxia and low ATP/AMP conditions, LKB1 activates AMPK, which phosphorylates TSC1/2 and Raptor components to reduce the level of mTORC1 activation. AKT is activated by LKB1, mTORC2 and PIP3, and the activated AKT inhibits TSC, which promotes the activated state of Rheb to activate mTORC1. After the preliminary activation of mTORC1, AKT continues to phosphorylate the inhibitory subunit PRAS40 to further activate mTORC1. mTORC1 activity is regulated in the above ways, which affects the expression of downstream genes such as SREBP1, PPARγ, HIF-α, ATF4, and PGC1α, influencing the intracellular ATP level and promoting the survival of hepatocellular carcinoma cells. Both LKB1 and AKT were the targets of neddylation, and after being modified by NEDD8, their stability was enhanced and the above activities were enhanced. Activated AKT also phosphorylates MDM2 to stabilize MDM2, promoting p53 ubiquitination. (ii) HuR stabilizing targeted mRNA. HuR modified by NEDD8 stays in the nucleus, and LKB1 also promotes the localization of HuR in the nucleus to protect it from proteasomal degradation to perform its function. HuR promotes the accumulation of MAT2A mRNA through post-transcriptional modification to promote the de-differentiation of hepatocytes. HuR stabilizes the HAUSP mRNA and controls apoptotic responses. LKB1, liver kinase B1; AMPK, AMP-activated protein kinase; AKT, protein kinase B; TSC, tuberous sclerosis protein complex; MDM2, mouse double minute 2; NEDD8, neural precursor cell expressed developmentally down-regulated protein 8; SREBP1c, sterol regulatory element binding protein 1c; PPARγ, peroxisome proliferator-activated receptor γ; mTORC1/2, mechanistic target of rapamycin (mTOR) complex 1/2; PIP3, phosphatidylinositol (3,4,5)-trisphosphate; HIF-α, hypoxia-inducible factor-alpha; ATF4, activating transcription factor 4; PGC-1α, peroxisome proliferator-activated receptor-gamma coactivator-1alpha; HuR, Hu antigen R; MAT2A, methionine adenosyltransferase 2A; HAUSP, herpesvirus-associated ubiquitin-specific protease.

In recent years, numerous non-cullin family proteins have been identified to be neddylated, and play important roles in the development of a wide range of tumors.^77^ Herein, we summarized the function of non-cullin proteins involved in HCC when they are neddylated, such as the kinases (LKB1, AKT), transcription factors (SREBP-1c, HBx), and RNA-binding proteins (HuR). In the context of overactivation of neddylation in HCC, the activities of these non-cullin substrates affect cellular processes, including substance metabolism and gene expression (Fig. 3 and Table 1).

### Neddylation of protein kinases

The protein kinase signaling pathway plays a vital role in HCC progression. Therefore, multiple kinases in HCC have been reported to be aberrantly regulated to promote the proliferation, invasion, and migration of tumor cells. Interestingly, the activity of some protein kinases has been reported to be regulated by the neddylation modification, including serine/threonine kinases LKB1 and Akt. Neddylaiton-regulated cell signaling affects the development of HCC.

Liver kinase B1 (LKB1), a protein kinase that directly phosphorylates and activates several kinases of the AMP-activated protein kinase (AMPK) family, senses the metabolic status in cells. LKB1 is highly expressed in HCC patients with poor prognosis. Moreover, LKB1 is found to be a target of neddylation, and then the neddylation modification would stabilize the LKB proteins.^78^ Thus, the overactivated neddylation process in HCC augments the protein level of LKB1 and triggers AMPK activities and their downstream signaling gene expression. For instance, under the condition of low O_2_ or low ATP/ADP, LKB1-AMPK will activate tuberous sclerosis protein complex and then the complex will inhibit the small GTPase Rheb (Ras homologue enriched in the brain); finally, both the two processes inhibited mTORC1 (mTOR- Raptor-mLST8-Deptor-PRAS40) activity. Given that mTORC1 acts as a cellular signal to influence the transcription of downstream genes, including SREBP1, PPARγ, HIF1α, activating transcription factor 4 (ATF4), and peroxisome proliferator-activated receptor-gamma coactivator-1alpha (PGC-1α), mTORC1 signaling pathway is responsible for energy metabolism. LKB1-AMPK axis down-regulates energy metabolic rate by inhibiting mTORC1 in HCC, which maintains HCC growth and survival under hypoxic stress conditions.^79^^,^^80^ Meanwhile, LKB1 mediates the phosphorylation of p53 and its cytosolic retention, which indicates the oncogenic effect of LKB1 in HCC.^81^^,^^82^ Considering that the transcriptional levels of the signaling genes related to LKB1 are correlated with survival outcomes among HCC patients, it can serve as a potential biomarker for clinical applications.^83^

AKT, also called protein kinase B, is activated by mTORC2 and PIP3 (phosphatidylinositol (3,4,5)-trisphosphate) in the PI3K/AKT/mTOR pathway. AKT can also be neddylated, and such modification increases the expression of AKT, which regulates HCC cell growth, proliferation, tumor cell angiogenesis, and metastasis by phosphorylating downstream substrates.^84^ In particular, AKT is considered a Warburg kinase to mediate aerobic glycolysis and metabolic reprogramming. In HCC, AKT phosphorylates and inhibits tuberous sclerosis protein complex to activate mTORC1 activity, and AKT phosphorylates the inhibitory subunit PRAS40 to further activate mTORC1 after the preliminary activation of mTORC1. By this means, AKT stimulates glycogen synthesis and increases the rate of aerobic glycolysis to regulate the apoptosis of HCC cells, which is complementary to the abovementioned regulation of cell metabolic state by LKB1.^85^ In addition, AKT inhibits MDM2 degradation by phosphorylating the Ser186 site of MDM2, which enhances its ubiquitinated degradation of p53 and exerts a potential oncogenic effect.^86^ One study found that LKB1 was also involved in inducing AKT activation in NASH-derived mouse HCC cells (SAMe-D), suggesting a crosstalk mechanism between LKB1 and AKT.

Except for the two protein kinases, several other protein kinases have been reported to be modified by NEDD8 in other cancer cells, such as TβRII and EGFR. Given the importance of such signaling pathways in the progression of HCC, the neddylation of these protein kinases could be an effective target for cancer treatment. Disruption of the neddylation process is a potential strategy to solve the drug resistance of tyrosine kinase inhibitor drugs.^87^, ^88^, ^89^

### Neddylation of transcription factors

Aberrant activation and/or inhibition of transcription factors cause significant changes in HCC-related gene transcriptional programs. In addition to the changes in DNA mutation or RNA transcription level, post-translational modifications also play important roles in regulating the activities of transcription factors and their associated tumorigenesis process. Neddylation-modified forms of transcription factors in HCC have been reported to be involved in the tumor process.

SREBP-1c is a transcription factor that regulates lipid homeostasis and pivotally promotes HCC cell proliferation, migration, and invasion. Typically, the stability of SREBP-1c is regulated by ubiquitination-mediated protein degradation. While the ubiquitin sites of SREBP-1c can also conjugate with a NEDD8 molecule to compete with ubiquitin in the context of neddylation overexpression, neddylation of SREBP-1 stabilizes its protein levels and allows it to continue exerting its transcriptional activity and promotes the production of fatty acids and the development of metabolic-associated fatty liver disease-related HCC.^41^^,^^90^ Thus, due to enhanced neddylation, SREBP-1c is significantly overexpressed in HCC and can be used as a prognostic marker for HCC.^91^

HBV infection is a high-risk factor contributing to HCC development. HBV-encoded oncogene *X* protein (HBx) acts as a transcriptional activator to regulate the expression of target genes, which play a key role in the occurrence and development of HBV-associated liver cancer. It has been demonstrated that UBE2M (E2)-HDM2 (E3) mediates the neddylation of HBx, which specifically interferes with the ubiquitination in its HLH domain. Neddylation of HBx inhibits ubiquitin-dependent protein degradation and enhances its transcriptional activity. In addition, neddylated HBx acts as a cullin-associated factor (DCAF) receptor to bind to DDB1, thereby displacing one or more DCAFs that are conducive to HBV replication and stabilizing their function.^92^ The binding of DCAFs to DDB1 also recruits HBV-negative regulators for ubiquitylation degradation, which promotes the development of HBV-associated HCC.^35^^,^^93^ The neddylation inhibitor MLN4924 has been shown to inhibit HBV-induced hepatocarcinogenesis by reducing HBx stability.^94^

### Neddylation of HuR

HuR (Hu antigen R) protein is highly expressed in HCC and involved in the regulation of proliferation and differentiation of liver cells. HuR belongs to the Hu/elav proteins (human embryonic lethal abnormal vision) family, which are well-known RNA-binding proteins that selectively recognize and bind to AU-rich elements. The functions of HuR on regulating mRNA of oncogenes in HCC rely on its neddylation modification. As reported, MDM2 acts as the NEDD8 E3 ligase to HuR proteins, forming an MDM2-NEDD8-HuR regulatory framework in cells.^95^ HuR modification by NEDD8 protects HuR from proteasomal degradation, allowing HuR to exert post-transcriptional modification, which promotes the accumulation of methionine adenosyltransferase 2A (MAT2A) mRNA in hepatocytes, leading to the malignant transformation of hepatocytes, as well as exerts apoptosis-controlling effects by stabilizing herpesvirus-associated ubiquitin-specific protease (MAT2A) mRNA and activates hepatic stellate cells to promote hepatic fibrosis by stabilizing α-SMA mRNA.^87^^,^^96^ MDM2 and neddylated HuR could be potential diagnostic/therapeutic markers for HBV-associated HCC.

### Neddylation inhibitors and HCC therapies

Given the pivotal role of neddylation modification in HCC development, the neddylation cascade proteins become the emerging therapeutic targets for the treatment of liver cancers. Until now, multiple chemical neddylation inhibitors have been developed and exhibited survival benefits for HCC patients. Here, we summarized the current neddylation inhibitors and their effects on HCC (Table 2).

**Table 2:** Summary of inhibitors targeting neddylation.

Type	Name	Potency	Target protein	Cellular Activity
E1 Inhibitors	MLN4924	IC_50_ = 4 nM	NAE	Promoting SCF inactivation to induce HCC apoptosis. Reducing lipid synthesis by inhibiting neddylation of SREBP-1. Reducing HBV transcription factors and inhibits HBV replication by activating the MAPK signaling pathway.
	TAS4464	IC_50_ = 0.955 nM	NAE	Inhibiting SCF and leading to accumulation of Cyclins and inhibition of NF-κB, thereby inhibiting cancer cell growth and survival.
	ABP A3	IC_50_ = 2.5 μM	NAE UAE	Preventing the formation of aggregates that lead to the aggregation of misfolded proteins and promoting apoptosis in tumor cells. Leading to upregulation of substrates p53 and p21and then preventing cell proliferation.
	Rhodium (III) complex 1	IC_50_ = 0.3 μM	NAE	Blocking neddylation of Caco-2 cells, resulting in accumulation of CRL substrates IκBα and p27 to inhibit Caco-2 cell proliferation.
	M22	IC_50_ = 12.7 μM	NAE	Inhibiting AGS cell proliferation and inducing apoptosis in A549 cells.
	LP0040	IC_50_ = 0.76–3.29 μM	NAE UAE	Increasing levels of cell cycle inhibitors to inhibit proliferation of multiple cell lines and activates caspase 3 to induce apoptosis in A549 cells.
	Flavokawain A (FKA)	IC_50_ = 5 μM	NAE	Inhibiting SCF activity and promoting accumulation of p21 and p27 to induce apoptosis.
	Flavokawain B (FKB)	IC_50_ = 986.1 nM	NAE	Inhibiting SCF activity and promoting accumulation of p21 and p27, inducing apoptosis.
	Gartanin	IC_50_ = 10.33 μM	NAE	Blocking neddylation to upregulate p27 and p21, and inhibiting tumor proliferation.
	Piperacillin	IC_50_ = 1 μM	NAE	Promoting the accumulation of the downstream protein p27 and inhibiting the proliferation of tumor cells.
	Mitoxantrone	EC_50_ = 1.3 μM	NAE	Increasing p53 transcriptional activity and inducing apoptosis in colorectal adenocarcinoma cancer cells.
	Candesartancilexetic	IC_50_ = 16.43 μM	NAE	Blocking the neddylation of Cullin1 and inducing apoptosis in A549 cells.
E2 Inhibitors	DC-1	IC_50_ = 1.2 μM	DCN1	Inhibiting neddylation of Cullin3.
	DC-2	IC_50_ = 15 nM	DCN1	Inhibiting neddylation of Cullin3 and tumor cell invasion.
	WS-383	IC_50_ = 11 nM	DCN1	Inhibiting neddylation of Cullin1 and Cullin3, inducing accumulation of p21, p27 and Nrf2.
	DN-2	IC_50_ = 9.6 nM	DCN1	Inhibiting neddylation of Cullin3.
	Compound 27	IC_50_ = 200 nM	DCN1	Inhibiting neddylation of Cullin1 and Cullin3.
	Compound 40	IC_50_ = 100 nM	DCN1	Inhibiting neddylation of Cullin1 and Cullin3.
	DI-591	Ki = 12 nM	DCN1	Inhibiting neddylation Of Cullin3 and promoting the accumulation Of NRF2.
	DI-404	Kd = 6.7 nM	DCN1	Inhibiting neddylation of Cullin3.
	DI-1548	Ki ＜ 1 nM	DCN1	Inhibiting neddylation of Cullin3 and promotes the accumulation of Nrf2.
	DI-1859	Ki ＜ 1 nM	DCN1	Inhibiting neddylation of Cullin3 and promoting the accumulation of NRF2.
	Arctigenin	IC_50_ < 2.5 μM	UBE2M	Blocking neddylation of CUL1-4. Inhibiting Wnt/β-catenin signaling pathway, inhibiting metastasis and invasion of HCC.
	HA-9104	IC_50_ = 49 μM	UBE2F	Inhibiting neddylation of Cullin5 to promote apoptosis in lung cancer cells.

NAE: Nedd8-activating enzyme; UAE: Ubiquitin-activating enzyme; SCF: Skp1-Cdc53/CUL1-F-box protein, a E3 Ub ligase complex; DCN1: Defective in Cullin Neddylation 1, a E3 ligase; UBE2M: Ubiquitin-conjugating enzyme E2 M; UBE2F: ubiquitin-conjugating enzyme E2 F.

### E1 inhibitors

NAE is the only NEDD8-activating enzyme in mammalian cells, therefore inhibition of NAE leads to the loss of entire neddylation modification of both cullin and non-cullin proteins, resulting in the aberrant cellular processes of DNA replication, DNA damage repair, apoptosis, and cell senescence. Given the overactivated neddylation pathway in HCC, NAE becomes a significant therapeutic target for HCC as well as many other tumors.^97^ To date, several covalent NAE inhibitors have been developed and entered clinical trials for tumor treatments. Among them, MLN4924, also known as pevonedistat, is the first NAE inhibitor entering clinical trials.^98^ It exhibits promising anti-tumor effects in human liver cancer xenotransplantation models and I-III clinical trials. Based on the current studies, the anti-HCC mechanism of MLN4924 relies on several cellular pathways. Briefly, MLN4924 blocks the cullin neddylation and then suppresses SCF E3 activation, which leads to the accumulation of its substrates p21, p27, and IκB, which induces apoptosis of hepatoma cells and inhibits HCC development.^99^ Besides, the Deptor, another SCF E3 substrate mediating mTOR inactivation, is stabilized and accumulated under MLN4924 treatment, which triggers the protective autophagy process. Therefore, combining autophagy inhibitors with MLN4924 can enhance its efficacy in promoting the apoptosis of HCC cells.^100^ Additionally, MLN4924 can reduce lipid synthesis by inhibiting the neddylation of SREBP-1 and therefore inhibit HCC progression. Recent studies also revealed that MLN4924 promotes the activation of the MAPK signaling pathway, and activated ERK1/2 inhibits the expression of several transcription factors required for HBV replication (HFN1α, HFN4α, and C/EPBα), thereby exerting anti-HBV activity and inhibiting the development of HBV-induced HCC.^94^ Inhibition of neddylation with MLN4924 is also reported to sensitize liver cancer cells to sorafenib (the first-line drug for advanced liver cancer) reducing their resistance.^23^

In addition, several other NAE-targeted inhibitors have been reported and investigated in cancer treatment. For instance, TAS4464 owns the IC50 value as low as 0.955 nmol/L against NAE for the Ubl thioester transfer activity, much lower than the IC50 value of 10.5 nmol/L for MLN4924. Thus, TAS4464 shows greater inhibitory effects to reduce substrate neddylation and leads to SCF inactivation with quicker efficacy and longer duration compared with MLN4924.^101^ Besides, ABP A3 owns dual inhibitory functions for both ubiquitin-activating enzyme (UAE) and NAE activities to block the neddylation process, leading to p21 up-regulation and tumor apoptosis.^102^ M22, cyclometallated rhodium (III) complex [Rh (ppy)2 (dppz)], is a reversible NAE inhibitor, which also effectively blocks NEDD8-mediated CRL activation. It exerts anti-tumor activity by promoting the accumulation of substrate IκBα and p27 in Caco-2 cells.^103^ M22 is a novel reversible NAE inhibitor that effectively inhibits the proliferation of various cancer cell lines at lower concentrations such as AGS cells while inducing apoptosis of A549 cells.^104^ LP0040 is rationally designed based on M22 as a non-nucleoside NAE/UAE dual inhibitor that can inhibit the proliferation of various cell lines including AGS cells while up-regulating the level of cell cycle inhibitors and activating caspase 3. Some natural products have been screened for their inhibitory activities of NAE. For example, flavokawain A (FKA) docks into the ATP binding pocket of NAE complex and inhibits cullin-1 neddylation and SKP2 degradation by suppressing SCF-SKP2-type ubiquitin ligase complexes' activity.^105^ Flavonoid B (FKB), isolated from kava root extract, also directly binds to NAE and has strong apoptotic activity against various cancer cell lines including prostate cancer, similar to FKA.^106^ The 4-isopentenyl flavonoid gartanin extracted from purple mangosteen fruit demonstrates analogous functionality to FKA and FKB.^107^ Besides, some FDA-approved drugs, such as candesartan ester drugs, mitoxantrone, and piperacillin, have been shown to competitively non-covalently bind to ATP sites on NAE and inhibit NAE activity.^108^, ^109^, ^110^

### E2 inhibitors

Two NEDD8-conjugating enzymes (E2s) have been identified in mammalian cells, including UBE2M (UBC12) and UBE2F. GEO database analysis revealed that high expression of UBE2M was associated with multiple metastasis, microvascular infiltration, and immune evasion in HCC patients, leading to a worse overall survival.^111^, ^112^, ^113^ Thus, the drugs targeting E2s to disrupt the interaction between E2 and E1 or E2 and E3, show a great effect in tumor suppression.^114^

UBE2M mediates the neddylation of both cullin and non-cullin proteins in HCC and several targeted drugs have been developed. Several inhibitors have been designed to target cullin neddylation protein (DCN1) to disrupt its interaction with UBE2M, reducing the development of HCC. Based on their original compounds, a series of small molecules have been developed to target the interaction of DCN1 with UBE2M. Firstly, five pyrimidine-based inhibitors have been synthesized and improved, including DC-1 (IC50 = 1.2 μM), DC-2 (IC50 = 15 nM), WS-291 (IC50 = 5.82 nM), WS-383 (IC50 = 11 nM), and DN-2 (IC50 = 9.6 nM).^115^^,^^116^ Besides, pyrazole-pyridone-based DCN1 inhibitors were improved with high inhibiting efficiency from the original compound (IC50 = 5.1 μM) to compound 27 (IC50 = 200 nM) and then compound 40 (IC50 = 100 nM) with more potency.^117^ Peptidomimetic small molecules own characterizations as high affinity and cell-permeability against UBE2M-DCN1 interaction, including DI591 (Ki = 12 nM), DI404 (Kd = 6.7 nM), and molecule DI-591; subsequently, DI1548 and DI1859 were further developed with more potent inhibitory ability.^118^^,^^119^ Besides, arctigenin, a natural product that inhibits UBE2M activity, decreases the neddylation of CUL1–4 in cells, which suppresses epithelial–mesenchymal transition in HCC cells and reduces the metastasis and invasion of tumors.^120^ Thus, inhibitors against UBE2M-DCN1 have been reported to affect downstream antioxidant, autophagy, and metabolism processes in hepatocytes and exhibit anti-tumor activity.

Compared with UBE2M, UBE2F has been relatively less studied. However, recent studies have shown that high expression of UBE2F is associated with poor prognosis for several types of human cancers.^18^^,^^113^ Moreover, oxidative stress caused by tumor chemotherapy and radiotherapy leads to the increase of UBE2F level, and anti-apoptotic drug resistance can be generated through the above process.^121^ Therefore, inhibition of UBE2F can not only inhibit tumor growth but also improve tumor sensitivity to radiotherapy and chemotherapy. UBE2F specifically interacts with RAX2/SAG to promote the neddylation of CUL5 and then mediates ubiquitination and degradation of cancer-associated proteins, including DIRAS2 and NOXA. Sun et al developed two small molecules HA-1141 and HA-9104 that targeted F56 and V30 of UBE2F-UBA3 binding pockets on the UBE2F surface, which then restrains the neddylation modification of CUL5.^122^, ^123^, ^124^, ^125^ These two UBE2F inhibitors could effectively induce apoptosis and G2/M arrest, which probably are further developed into anti-cancer reagents.^125^

Although most of the E2-targeted inhibitor drugs have not yet been used for HCC treatment, they could be a potent target drug pool to further investigate HCC therapy.

### E3 inhibitors

So far, few publications have reported the specific inhibitors for NEDD8 E3 ligase used in HCC or other cancers. Given that most NEDD8 E3 ligases show ubiquitin ligase activity, the dual roles in one protein make it hard to specifically block their active site of NEDD8 ligase, rather than ubiquitin ligase. With the development of powerful tools to solve protein structures with high resolution, more specific druggable sites for NEDD8 E3 would be targeted to design inhibitors for the therapy of HCC or other cancers.

## Concluding remarks

Neddylation, as the essential post-translational modification in cells, regulates a variety of important life activities in cells through the modification of substrates. Overactivated neddylation appears in HCC and is closely associated with poor clinical prognosis. Numerous studies indicate that the dysfunction of neddylation is deeply associated with the progression of HCC proliferation, apoptosis, and angiogenesis. Thus, the neddylation cascade could be a new potent target for early diagnosis and treatment of HCC. Clinical trials have indicated the potent clinical efficacy of specific neddylation inhibitors, such as MLN4924 and TAS4464. With the development of druggable sites on the proteins in the neddylation cascade, more corresponding compounds can advance to clinical trials and be used for HCC treatment in the future.

## Funding

This work was supported by the 10.13039/501100007129Natural Science Foundation of Shandong Province, China (No. ZR2021QC030, ZR2022LZL006) and the Innovation Project of Shandong First Medical University.

## CRediT authorship contribution statement

**Wenxin Wu:** Conceptualization, Writing – original draft. **Xuanyi Wang:** Writing – original draft. **Ruijie Ma:** Writing – original draft. **Shuhong Huang:** Conceptualization, Writing – original draft, Writing – review & editing. **Hongguang Li:** Writing – original draft, Writing – review & editing. **Xinxing Lyu:** Conceptualization, Formal analysis, Funding acquisition, Writing – original draft, Writing – review & editing.

## Conflict of interests

The authors declared no competing interests.
